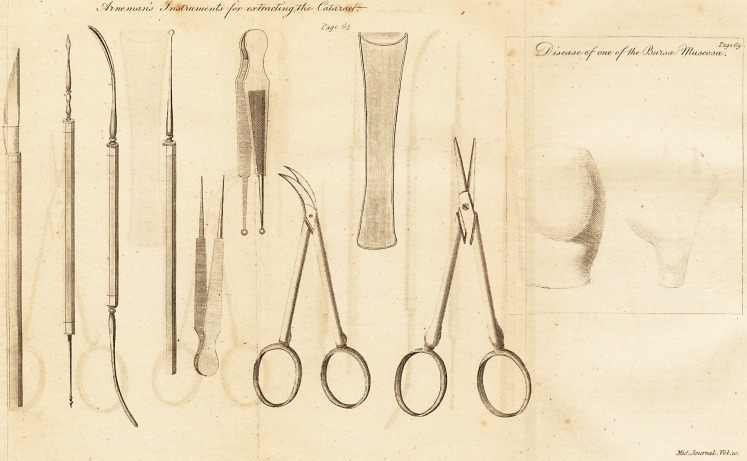# Professor Arneman's Instruments for Extracting the Cataract

**Published:** 1803-07-01

**Authors:** 


Arneman's Instruments for extracting the Cataract.

### Professor ARNEMAN'S INSTRUMENTS *for extracting the* CATARACT.

[ With an Engraving. ]

THE extraction of the cataract is undoubtedly an operation which requires the greatest nicety, skilfulness, and steadiness of hands in the operator, as also a construction of the instruments most accurately adapted to the delicacy of that operation. It cannot be denied, that this branch of operative surgery is executed with particular success by several oculists, and other medical men of Germany; and however inferior they may consider themselves to the English surgeons in many parts of surgical operations, they think themselves entitled to claim, if not the superiority, at at least the same merit and dexterity in performing this delicate operation. Amongst the medical gentlemen of that country, who have acquired great celebrity in this respect, Prof. Arneman, of Gottingen, deserves to rank high, as not only the great number of patients who have regained the happiness of sight through his skilful hands and prudent management, are the surest pledges of his abilities, but also several improvements which he has made on the instruments and modus operandi, manifestly shew how intimately he is acquainted with the nature of that operation. We feel particular satisfaction in communicating to our readers an abstract of a chapter from Prof. Arneman's treatise on the diseases of the eye, which make the second volume of his useful System of Surgery, a judicious compendium of that art, not long ago published at Gottingen.

" When we consider, the author says, the various forms and the great number of instruments invented for the operation of the cataract, and particularly for the incision of the cornea, we may be led to think either that the figure and structure of the cornea is differently formed in the individuals of different nations, or, this being improbable, that this part of an operation, which requires so much nicety and skilful management of the hand must have often been performed in a very rude manner, and we may thus account for the ill success with which this operation is so frequently attended. On comparing the knives invented at the first period, when the extraction of the cataract was established by Daviel; the narrow instruments of La Faye, Palucci, Sharp, Warner, with the broad lancet of Mr. Wathen in our present times; on reviewing the bicuspid knives of Poyet and Siegwart; the knives of Boranger and Casamata; the serpentine knife of Tellier; the long and pointed knife of Siegerist; the small pen-knife of Muller; the knives of Wentzel, Richter, Lobstein, Bell, Mursinna, and others; the long knife of Barth and Beer; and the triangular pointed knife of Santerelli; it is hardly possible to imagine how so small a part, having only a few lines in circumference, as the surface of the cornea, could have admitted of such a variety of inventions, without their proving for the most part prejudicial to the operation.

" It is obvious to remark, that a proper and convenient form, as well also as the size of the instrument, must in a great measure influence the good or ill success of the operation. An instrument which is too narrow will make an incision in the cornea not sufficient for the crystalline lens lens to pass through, without its being broken to pieces, or without the mucous coat being stript off and thus remaining in the eye, which renders it necessary to enlarge the incision, whereby the sight of the patient may be injured either by the scar of the wound, or by a cataracta secundaria. Another objection may be urged against the broad instruments, which by making the incision too wide at once, suffer the cataract to pass out too suddenly, whence it not unfrequently happens, that by its being protruded with some violence by the contraction of the bulb of the eye, a prolapsus of the vitreous humour is produced, and in this manner the interior parts of the eye become more liable to inflammation. The instruments formed in the shape of a lancet have the inconvenience of making an irregular incision; the exit of the instrument, when used for cutting the cornea, not being situated exactly opposite in the same line where it had entered the cornea, which will make the opening of the cornea appear in an inclined direction. The exterior angle of the cornea suffers besides a greater pressure from the knife; and we may frequently observe, that the cornea, from the incision not being quite regular, nor equally wide in every dimension, will be greatly injured, and not be brought to heal by simple union, but begin to ulcerate in a more or less degree; a circumstance by which an opacity is often occasioned, to the greatest distress and disadvantage of the patient as well as of the operator."

Induced by these considerations, and guided by his own experience, Prof. Arneman has found that knife to be best calculated for the incision of the cornea, which is engraved in the annexed plate, fig 1; and he is inclined to ascribe, in some measure, to this improvement of the knife, the success with which his operations were subsequently attended.

### DESCRIPTION OF THE PLATE.

Fig. 1. Represents the knife of Prof. A. for the incision of the cornea, made by the justly celebrated Mr. Savigny, of London, after a pattern sent to him from Gottingen. The back of the knife is exactly in a straight line; its point is narrow, sharp, and gradually increasing in breadth; it is convex on both sides. It passes through the cornea with great ease, and never permits the aqueous humour to run out, before it has made its way through the camera anterior of the eye, and the incision becomes regularly semicircular. The blade of the knife measures, at its broadest part, about three lines, which is jusly adapted to let the lens pass through easily. The handle is octogonal, and a little flattened or compressed.

Fig. 2. A broad needle, shaped like a lancet, which may be used for couching the cataract. Prof A. however, sometimes employs this instrument strument for opening the capsule of the lens. To the inferior part of the handle is adapted a small screw, somewhat resembling a cork-screw. This instrument was occasionally invented by Prof. Arneman in a case of operation, where the crystalline lens adhered pretty strongly to the iris with almost its whole circumference. This instrument however being screwed into the middle of the cataract, it was easily detached; and being gently moved in all directions, it came away with great ease. With this instrument the lens is easily extracted, together with the capsule; but if the cataract is rather dissolved and of a pulpy consistency, it is difficult, if not impossible to fix the screw. This inconvenience, however, may in a great measure be remedied by the screw being made double.

Fig. 3. Represents at the upper part a small spoon like that of Daviel. It will be found of great use for taking away mucous particles and filaments, also small detached pieces of the cataract, &c. At the lower end Mr. A. has adapted a similar instrument without a canula, flat and smooth on both sides. It proves very serviceable for lifting up the part of the cornea which is cut, and it is likewise of great use in assisting the passage of the cataract, if it is generally pressed on the inferior part of the bulb of the eye, as this cannot be done so conveniently and nicely with the finger.

Fig. 4. Represents a small, semi-circular hook, which Prof. A. makes use of for the purpose of taking out the cataract, a method which may be recommended as a very useful improvement. After having made the incision of the cornea, Mr. A. without opening the capsula of the lens, applies this small hook through the pupil, with the point turned outwards. Turning the instrument in the pupil, he fixes the point of the hook from below in the centre of the cataract; by which means, without doing the least injury to the eye, either by pressure or irritation, the cataract being pulled out gently and generally along with the capsula. By this method the eye is guarded against a cataracta secundaria, formed by the opacity of the capsula.

Fig. 5 and 6. Two pair of nippers or small pincers for extracting filaments, for which purpose the pointed one seems to be particularly calculated.

Fig. 7. A broad hook for lifting up the upper eye-lid during the operation.

Fig. 8. A curved and concave bladed pair of scissars. The figure will not give a clear idea of this instrument without being explained by a description. The instrument is merely represented with a curved blade, but it is very essential that the blade be made concave. Prof. Arneman has invented this instrument for the operation of the cataracta secundaria. It is generally agreed, that the cataracta secundaria is beyond the limits of the art; but in some cases, this gentleman has succeeded in removing this disagreeable complaint. The secondary cataract is often caused by the capsula lentis remaining in the eye and becoming opaque; sometimes some mucous matter, detached from the cataract, occasions this defect. It however more frequently originates from the extravasation of lymph, by which the iris is obstructed, in consequence of an inflammation of the interior parts of the eye, produced in rheumatic or gouty habits, and by any injury sustained during the operation. In this case, after having opened the cornea a second time, he makes a circular incision in the iris with the above scissars, and in this manner the patient's sight is restored.

Fig. 9. A pair of straight scissars for different purposes, particularly if the cataract adheres to filaments, which are to be cut through. For the operation itself, they are of no immediate use, in which Prof. A. condemns, on the whole, all sorts of scissars as useless and prejudicial.

Prof. A. has communicated in his Treatise on the Diseases of the Eye, several interesting and new methods of treatment, which we shall lay before our readers at some future period.

## Figures and Tables

**Figure f1:**